# Application of a practice-based approach in variable selection for a prediction model development study of hospital-induced delirium

**DOI:** 10.1186/s12911-023-02278-1

**Published:** 2023-09-13

**Authors:** Urszula A. Snigurska, Sarah E. Ser, Laurence M. Solberg, Mattia Prosperi, Tanja Magoc, Zhaoyi Chen, Jiang Bian, Ragnhildur I. Bjarnadottir, Robert J. Lucero

**Affiliations:** 1 College of Nursing, Department of Family, Community, and Health Systems Science, University of Florida, 1225 Center Drive, PO Box 100197, Gainesville, FL 32610 United States of America; 2 College of Public Health and Health Professions & College of Medicine, Department of Epidemiology, University of Florida, 2004 Mowry Rd, Gainesville, FL 32610 United States of America; 3 Geriatrics Research, Education, and Clinical Center (GRECC), North Florida/South Georgia Veterans Health System, 1601 SW Archer Rd, Gainesville, FL 32608 United States of America; 4 College of Medicine, University of Central Florida, 6850 Lake Nona Blvd, Orlando, FL 32827 United States of America; 5 Clinical and Translational Science Institute (CTSI), Integrated Data Repository Research Services, University of Florida, 3300 SW Williston Rd, Gainesville, FL 32608 United States of America; 6 College of Medicine, Department of Health Outcomes & Biomedical Informatics, University of Florida, 2004 Mowry Rd, Gainesville, FL 32610 United States of America; 7 School of Nursing, University of California Los Angeles, 700 Tiverton Ave, Los Angeles, CA 90095 United States of America

**Keywords:** Candidate predictor, Delirium, Expert judgment, Practice-based approach, Prediction model, Variable selection

## Abstract

**Background:**

Prognostic models of hospital-induced delirium, that include potential predisposing and precipitating factors, may be used to identify vulnerable patients and inform the implementation of tailored preventive interventions. It is recommended that, in prediction model development studies, candidate predictors are selected on the basis of existing knowledge, including knowledge from clinical practice. The purpose of this article is to describe the process of identifying and operationalizing candidate predictors of hospital-induced delirium for application in a prediction model development study using a practice-based approach.

**Methods:**

This study is part of a larger, retrospective cohort study that is developing prognostic models of hospital-induced delirium for medical-surgical older adult patients using structured data from administrative and electronic health records. First, we conducted a review of the literature to identify clinical concepts that had been used as candidate predictors in prognostic model development-and-validation studies of hospital-induced delirium. Then, we consulted a multidisciplinary task force of nine members who independently judged whether each clinical concept was associated with hospital-induced delirium. Finally, we mapped the clinical concepts to the administrative and electronic health records and operationalized our candidate predictors.

**Results:**

In the review of 34 studies, we identified 504 unique clinical concepts. Two-thirds of the clinical concepts (337/504) were used as candidate predictors only once. The most common clinical concepts included age (31/34), sex (29/34), and alcohol use (22/34). 96% of the clinical concepts (484/504) were judged to be associated with the development of hospital-induced delirium by at least two members of the task force. All of the task force members agreed that 47 or 9% of the 504 clinical concepts were associated with hospital-induced delirium.

**Conclusions:**

Heterogeneity among candidate predictors of hospital-induced delirium in the literature suggests a still evolving list of factors that contribute to the development of this complex phenomenon. We demonstrated a practice-based approach to variable selection for our model development study of hospital-induced delirium. Expert judgement of variables enabled us to categorize the variables based on the amount of agreement among the experts and plan for the development of different models, including an expert-model and data-driven model.

**Supplementary Information:**

The online version contains supplementary material available at 10.1186/s12911-023-02278-1.

## Background

Hospital-induced delirium among older adults has been estimated to cost the health care system in the United States from $38 to $152 billion every year [1]. Delirium is an acute neurocognitive syndrome and refers to a disturbance in attention and awareness with fluctuating intensity [2]. The sequelae of hospital-induced delirium include cognitive and functional decline [3]. Moreover, patients with hospital-induced delirium are at a two-times higher risk of death compared to patients without hospital-induced delirium [4].

At least 30% of hospital-induced delirium cases are preventable [5]. However, in order to prevent hospital-induced delirium, clinicians must be able to identify which patients are vulnerable and likely to develop delirium while they are hospitalized. Prognostic models that include potential predisposing and precipitating factors of hospital-induced delirium may be used to identify patients who are at risk of developing delirium and inform the implementation of tailored preventive interventions.

Many prognostic models of hospital-induced delirium exist specifically for older adults (Table S1 in the Supplementary Material) [6–20] and often target patients who are hospitalized in intensive care units [10–13, 16, 18–20]. However, existing models for the older patient population vary in the ability to discriminate between patients with higher and lower probability of developing hospital-induced delirium [21]. The accuracy of these models is limited due to methodological shortcomings during model development and validation, including how candidate predictors and outcomes are operationalized [21].

*Predictors* are variables that are used to create a prognostic model [22]. *Candidate predictors* refer to all variables that are evaluated for their association with an outcome regardless of whether they are included in the final model [22]. It is recommended that candidate predictors are selected on the basis of existing knowledge [22]. This includes knowledge from the scientific literature as well as clinical expertise [23]. In our larger study, we are developing prognostic models of hospital-induced delirium for medical-surgical older adult patients using structured data from administrative and electronic health records. The purpose of this article specifically is to describe the process of identifying and operationalizing candidate predictors using a practice-based approach. This a necessary step towards transparent reporting of a prediction model study [24].

## Methods

All of our methods were carried out keeping in mind the Transparent Reporting of a Multivariable Prediction Model for Individual Prognosis or Diagnosis (TRIPOD) Statement. [24] Our methodological process included the following steps: (1) review of existing literature, (2) expertise-driven, manual extraction of variables that were used as candidate predictors in step 1, (3) expert judgment of variables that were extracted in step 2, (4) mapping of the variables from step 3 to the administrative and electronic health records, and development of operational definitions for the variables (operationalization of the variables).

### Step 1: Review of existing literature

In our previous work, we conducted a systematic review of the literature to identify research designs and analytic methods that had been used to develop and validate prognostic model(s) of hospital-induced delirium for adult patients. The protocol for this review, including database-specific syntax, is available under registration number CRD42020218635 (version 03 December 2020) in the International Prospective Register of Systematic Reviews “PROSPERO”. We searched the Cumulative Index to Nursing and Allied Health Literature (CINAHL), Medical Literature Analysis and Retrieval System Online (MEDLINE), American Psychological Association (APA) PsycInfo, and Web of Science Core Collection on August 22, 2020, to identify relevant studies. Details about the eligibility criteria and selection process are provided under “Additional Information about Step 1: Review of Existing Literature” in the Supplementary Material. A total of 42 studies were included, specifically 34 model development-and-validation studies and 8 validation-only studies (Figure S1 in the Supplementary Material).

### Step 2: Expertise-driven, manual extraction of variables

The aforementioned review was used to extract variables. However, the inclusion criteria in the 03 December 2020 version (see “Eligibility Criteria” under “Additional Information about Step 1: Review of Existing Literature” in the Supplementary Material) were not specific to the medical-surgical older adult patient population, but included adult patients regardless of unit type. We still decided to use all of the 34 model-and-validation studies that were included in the original review for the variable extraction (Table S1 in the Supplementary Material). While we understand that risk factors of delirium may vary from patient population to patient population, there may also be some risk factors that are universal across healthcare settings. Furthermore, having screened 4,312 records for the review (Figure S1 in the Supplementary Material), we observed that the type(s) of unit were rarely specified in the articles. Because we did not want to leave out any potentially significant risk factors solely on the basis of limited information, we decided to keep our initial pool of variables broad and narrow it down in further steps of variable selection.

We referenced the “Candidate Predictors (Or Index Tests)” domain of the Checklist for Critical Appraisal and Data Extraction for Systematic Reviews of Prediction Modelling Studies to check for the presence of relevant information about the variables that had been used as candidate predictors during model development (Table S2 in the Supplementary Material). [25] A researcher of Nursing Sciences with four years of experience as a registered nurse in the acute care setting (U.A.S.) manually extracted clinical concepts and their associated measurements (operational definitions). For example, both the concept “dehydration” and its measurement “blood urea to nitrogen ratio” were extracted from Carrasco et al.’s (2014) study [6] . Clinical concepts with missing measurements were not extracted, for example, “medical data”, “medication”, and “preoperative laboratory values”. [26].

### Step 3: Expert judgment of variables

After we had identified and extracted the variables from the model development-and-validation studies, we created a list of these variables in a Microsoft Excel spreadsheet. In the spreadsheet, we programmed a dropdown with “Yes” and “No” answer options for each variable. We consulted experts from a multidisciplinary Iatrogenic Conditions Task Force (ICTF) (UF IRB #201900208) who judged whether each variable was associated with the development of hospital-induced delirium. The ICTF members included six registered nurses (three with a master degree, two with a bachelor degree, and one with a doctoral degree), two physical therapists (both with a doctoral degree), and one internal medicine physician.

We distributed the spreadsheet by e-mail and instructed the ICTF members to answer “Yes” or “No” to the question, “Do you consider this to be associated with delirium?”, for each variable. The exact wording of the e-mail is provided in the Supplementary Material (Box S1). The variables in the spreadsheet were ordered alphabetically not to potentially suggest that the variable above was more important than the variable below. We further encouraged the ICTF members to add their own ideas by including, “Would you like to add anything else? Please add in the column to the right.”, at the end of the spreadsheet.

Our goal was to generate true judgments from the ICTF members. We purposely did not allow for ambiguity in the survey by, for example, adding “Maybe” as an answer option. We already knew that the variables were “maybe” associated with hospital-induced delirium, because we had extracted them from the literature. In the case when an ICTF member selected neither “Yes” nor “No” for a variable, we assumed that the ICTF member had insufficient information to affirm that the variable was associated with hospital-induced delirium. Therefore, a blank answer was considered a “No”. The ICTF members were blinded to the source of the variables, i.e., that they had been extracted from the literature.

After each ICTF member had filled out the Excel spreadsheet, we counted the number of ICTF members who answered “Yes” and calculated the percentage of agreement for each variable. We then ranked the variables from complete agreement (9/9) to no agreement (0/9). The variables with complete agreement specifically were considered the “expert” candidate predictors.

### Step 4: Mapping of variables to electronic health record system and development of operational definitions

We then explored whether and how each variable was represented in our local electronic health record (EHR) system, Epic, to be able to construct an operational definition of the variable for use in future studies that analyze data from the EHR system. A researcher of Nursing Sciences with four years of experience as a registered nurse in the acute care setting, specifically medical-surgical units in two different hospital systems (U.A.S.), in consultation with the ICTF members, reviewed all of the variables that had been extracted in step 2 and determined how each variable was represented in our Epic EHR system.

Reiterating the purpose of this study, we were only interested in the variables that could be operationalized using data that was (1) routinely collected in adult medical-surgical units, and (2) structured, such as coded data (for example, diagnosis codes), data entered in drop-down menus within thematic flowsheets, etc.

## Results

**Fig. 1:**
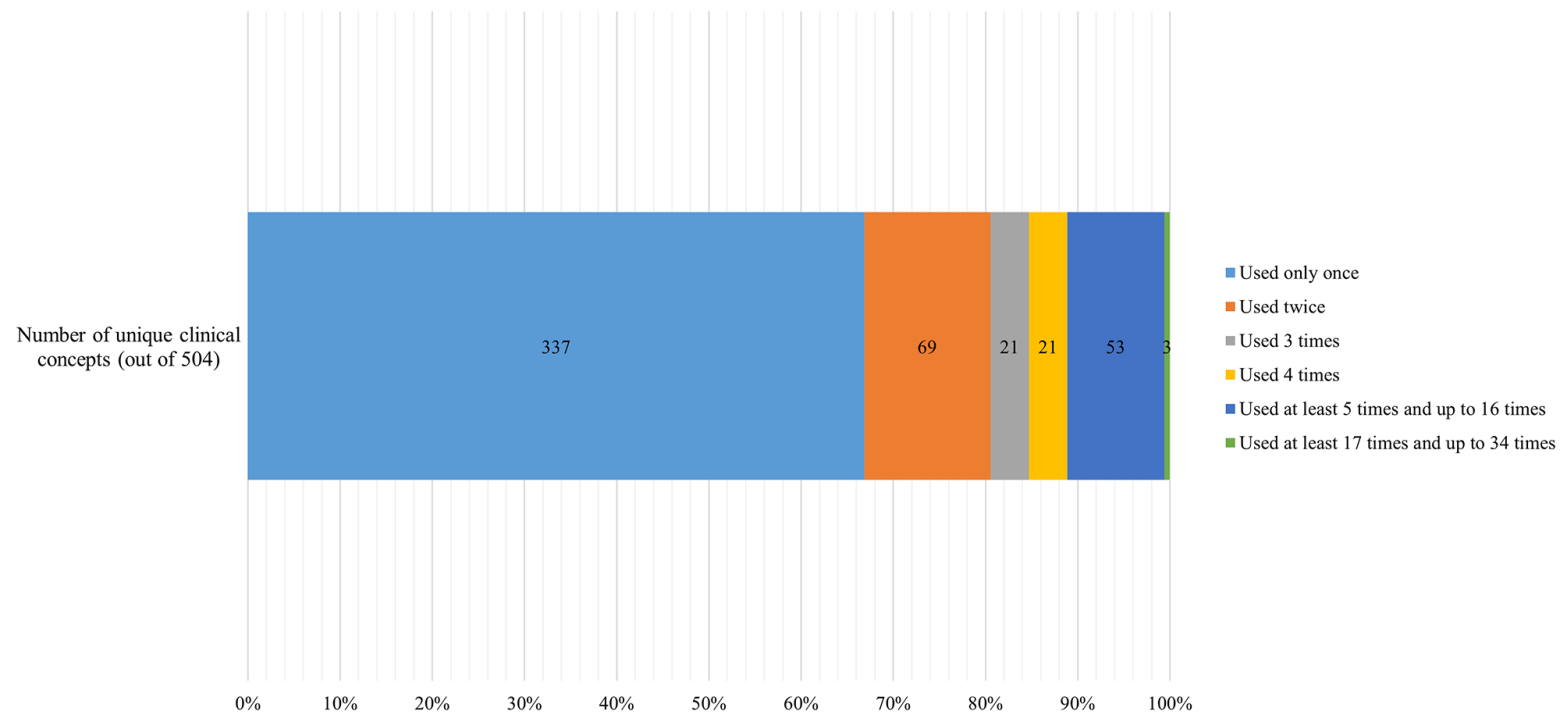
Appearance of Clinical Concept as Candidate predictor(s) across Prognostic Model Development-and-validation Studies of Hospital-induced Delirium. Note: This figure presents how many times a clinical concept was used as a candidate predictor across all of the prognostic model development-and-validation studies of hospital-induced delirium (n = 34). For example, 69 unique clinical concepts were used as candidate predictors twice.

We extracted 504 variables from 34 model development-and-validation studies. All of the studies are referenced and summarized in the Supplementary Material (Table S1). The complete list of the variables is also provided in the Supplementary Material (Table S3). Figure 1 presents how many times a variable was used as a candidate predictor across all of the studies. Two-thirds of the variables (337/504) were used as candidate predictors only once. One-third of the variables (167/504) were used as candidate predictors at least twice across all of the studies. The top three variables that were used as candidate predictors across at least half of the studies included age (31/34), sex (29/34), and alcohol use (22/34).

Table 1 presents the results of agreement among the ICTF members. Non-response was observed across 365 variables (72.4% of the 504 variables) and ranged from one ICTF member for 138 variables (37.8% of the 365 variables) to four ICTF members for one variable (0.27% of the 365 variables) with a median of two ICTF members. All of the ICTF members agreed that 47 variables were associated with hospital-induced delirium.

**Table 1:** Cumulative Number of Variables for Each Number of ICTF Members who Answered “Yes”

Number of ICTF Members who Answered “Yes” (Out of 9)	Number of Variables	Cumulative Number of Variables
9 (100%)	47	47
8 (89%)	40	87
7 (78%)	52	139
6 (67%)	66	205
5 (56%)	73	278
4 (44%)	85	363
3 (33%)	66	429
2 (22%)	55	484
1 (11%)	17	501
0 (0%)	3	504

*Note*. ICTF = Iatrogenic Conditions Task Force.

Three variables with complete agreement (physical restraints, sedation status, and withdrawal) make up our operational definition of hospital-induced delirium that is the outcome in our larger model development study. These variables were excluded from our pool of candidate predictors to decrease bias in the outcome measure [22].

Other four variables with complete agreement were not considered further in this study, because they were exclusively captured as *un*structured (text) data in our Epic EHR system. These variables were most accurately and reliably documented in clinicians’ narrative notes and included (1) duration of anesthesia, (2) physical status (measured with the American Society of Anesthesiologists Physical Status Classification), (3) severity of acute illness (measured with the Acute Physiology and Chronic Health Evaluation), and severity of stroke (measured with National Institutes of Health Stroke Scale).

Table 2 presents the list of the remaining 40 “expert” candidate predictors of hospital-induced delirium. Notably, 23 or 57.5% of them were included in the final prognostic models of hospital-induced delirium in the model development-and-validation studies based on statistical evaluation. These variables (empirical predictors) are denoted by asterisks in Table 2.

**Table 2:** “Expert” Candidate Predictors of Hospital-induced Delirium (n = 40)

Administrative	Diagnosis	Laboratory Test	Medication	Nursing Assessment	Surgery
Age* Length of hospital stay* Length of intensive care unit stay*	Alcohol use* Alzheimer’s disease* Anxiety* Cerebral edema Dementia* Depression* History of delirium* Mental disorder* Neurologic disease* Post-surgical complications Psychosis Respiratory disease Respiratory failure* Respiratory infection Sepsis Shock Sleep disorder Substance use Trauma* Urinary tract infection*	Prolonged bleeding (due to overanticoagulation and/or procedure)	Fentanyl Minerals and electrolytes* Opioids Propofol* Psychotherapeutic agents* Sedatives*	Cognitive status* NPO state Pain* Psychological status Risk of falls Sleep deprivation*	General anesthesia* Neurosurgery Surgery* Trauma surgery

*Note*. The candidate predictors are organized by operational category based on how they were most accurately and reliably represented in our local EHR system, Epic. EHR = electronic health record; NPO = nil per os (“nothing by mouth”).

* Empirical predictors of hospital-induced delirium (candidate predictors that were included in the final prognostic models of hospital-induced delirium in the model development-and-validation studies based on statistical methods (see Table S1 in the Supplementary Material for the list of studies)).

Based on how they were most accurately and reliably represented in our Epic EHR system, all of the variables that were extracted in step 2, including the variables that were later determined to be “expert” candidate predictors based on step 3, were divided into six operational categories, including administrative, diagnosis, laboratory test, medication, nursing assessment, and surgery (Table 2). Table 2 lists the “expert” candidate predictors only.

The “administrative” category includes variables that can be directly pulled from administrative databases (for example, age at encounter) or operationalized using multiple administrative data elements that have to be manipulated first (for example, the variable “length of hospital stay” is operationalized by subtracting the date of discharge from the date of admission).

The “diagnosis” category includes variables that can be operationalized using diagnosis codes based on the International Statistical Classification of Diseases and Health Related Problems, Clinical Modification (both the legacy ninth revision and the current tenth revision).

The “laboratory test” category includes values of laboratory tests that are coded using the Logical Observation Identifiers Names and Codes.

In addition to specific medications (for example, morphine sulfate), the “medication” category includes multiple medications that are grouped based on the classification that Epic uses for the Medication Administration Record.

The “nursing assessment” category includes variables that can be operationalized using (structured) data (that excludes free-text entries) from nursing flowsheets. Examples of nursing flowsheets that are routinely used by the medical-surgical nursing staff in our hospital system include the flowsheets under the “ADT Navigators” (“ADT” stands for “Admission, Discharge, Transfer”) and “Avatar” (flowsheets specific to airways, drains, lines, and wounds, so anything that is connected *to*, *in*, or *on* a patient), “Daily Care”, “Intake/Output”, “Pain Assessment”, “Simple Assessment”, and “Vital Signs Simple”. Various other flowsheets can be used for ad-hoc assessments (“Blood Transfusion”, “Restraints”, etc.).

The “surgery” category includes variables that can be operationalized using procedure codes based on the Current Procedural Terminology.

## Discussion

We completed the first step of variable selection for the development of a prognostic model [23]. Based on the synthesis of data from the literature review, that resulted in the extraction of 504 unique variables that had previously been used as candidate predictors of hospital-induced delirium, and subsequent consultation with the multidisciplinary ICTF, we identified 484 clinical concepts that had been judged to be associated with the development of hospital-induced delirium by at least two ICTF members. Forty clinical concepts were determined to be “expert” candidate predictors of hospital-induced delirium based on the complete agreement among the nine ICTF members.

The most prominent finding is that we extracted over 500 unique candidate predictors of hospital-induced from the review of 34 separate studies. This number is conservative because we did not extract candidate predictors that had not been operationally defined in the articles. Such a heterogeneity among candidate predictors of hospital-induced delirium suggests a still evolving list of factors that contribute to the development of this complex phenomenon. The finding also supports the recommendations from domain experts to include clinical expertise in the selection of candidate predictors for prediction model development [23].

Consistent with these recommendations, we engaged the ICTF in our process of variable selection to triangulate the findings from our literature review and identify “clinically meaningful” candidate predictors of hospital-induced delirium. At least two ICTF members agreed on 96% of the variables that had been extracted from 34 different prognostic model development-and-validation studies of hospital-induced delirium. This finding suggests that close to all existing candidate predictors of hospital-induced delirium may be clinically meaningful. While these candidate predictors may be clinically meaningful, they may be, however, correlated with each other, and, therefore, redundant. In the second step of variable selection for the development of a prognostic model, statistical methods are used to determine which candidate predictors are important and which candidate predictors can be discarded without compromising the model performance [23]. If a number of candidate predictors is highly correlated with each other, only one candidate predictor may ultimately be selected for inclusion in the final prediction model.

Our process of variable selection has enabled us to categorize the variables based on the amount of agreement among the ICTF members and use these categorized variables in different models. In this study, our purpose was to identify candidate predictors that would be used for an expert-driven prognostic model of hospital-induced delirium. To feel confident in our future model, we decided to use candidate predictors that reached complete agreement among the nine ICTF members.

However, there is a limitation to this decision. The complete agreement among the ICTF members is the most stringent criterion for the inclusion of a candidate predictor in a model and one that risks the possibility of leaving out other variables that may be associated with the development of hospital-induced delirium. This possibility is high in our case, because, on average, two ICTF members did not provide a response to almost three-fourths of the variables in the survey. So, we are missing roughly two responses for most of our variables, and even up to four for one variable. Hypothetically, if we had not imputed a “No” wherever there was a missing response, and the two missing responses, on average, had been “Yes” responses instead, we could have identified additional 92 “expert” candidate predictors of hospital-induced delirium, because 139 clinical concepts would have then been judged to be associated with the development of hospital-induced delirium by all of the nine ICTF members (moving from 7/9 to 9/9 agreement in Table 2).

Considering this limitation, it is important to emphasize that we have not discarded the variables with less than 100% agreement among the ICTF members. They have been operationalized according to the process that we described earlier and will be used in different models. One of our plans is to apply machine learning to all of the variables that have been extracted from the literature and develop a data-driven model and compare it to the expert-driven model.

Once variables have been selected, they must be operationalized. However, most knowledge-driven variables describe abstract clinical concepts that do not directly map to the EHR data [27]. The engagement of ICTF members in our process of variable selection was necessary to understand how the variables that we had extracted from the literature were represented in our Epic EHR system. The ICTF members were also critical in helping us select the most accurate and reliable representation of a variable if the variable appeared in multiple places in the EHR. For example, instead of using a procedure code for transfusion of blood or blood components (Current Procedural Terminology code 36430), we were advised to use data from the ad-hoc nursing flowsheet “Blood Transfusion” that is initiated every time a transfusion of blood or blood components is administered.

We believe that involvement of clinical experts, not only in the selection of variables, but also in their operationalization, is an innovative approach across model development studies, because it takes into consideration current limitations of data within the EHR systems. These limitations, such as low accuracy of a variable that is operationalized with some EHR data element(s), may introduce bias to models that are based on the EHR data, unless these limitations are corrected. Furthermore, this approach is user-friendly and may be one, collaborative way to improve future uptake of models that will be implemented in clinical practice.

Lastly, we want to comment on the involvement of nurses, specifically, in our process of variable selection. Most of the ICTF members were nurses, and they participated in both the expert judgment of variables as well as the mapping of variables to the EHR system and development of operational definitions. Nurses’ expertise is unique because it comes from nursing surveillance that is a process of an ongoing and purposeful acquisition, interpretation, and synthesis of patient data [28]. Nursing surveillance requires nurses to be aware of any, even subtle, changes in patient status and make connections among patient characteristics and outcomes [28]. This truly *practice*-based knowledge may be what narrows the gap in the missing knowledge about the etiology of delirium and contributes to the discovery of new predisposing and precipitating risk factors of this, and other, iatrogenic conditions. To leverage nurses’ practice-based knowledge, we advocate for a greater use of specifically nurse-generated data (for example, nursing flowsheets) in the operationalization of candidate predictors, just as we proposed in this study, by creating the “nursing assessment” operational category. This is because nurses, in the process of nursing surveillance, collect in real or almost real time an abundance of data on a variety of characteristics that may be important for accurate prognosis of hospital-induced delirium or other iatrogenic conditions. For example, in a study that only used nurses’ assessment data for the development of a prognostic model of hospital-acquired pressure injuries, the best prognostic model had the area under the receiver operating curve of 0.80 and this indicates “excellent” discrimination [29, 30].

### Limitations

There are a few important limitations to our study. First, nearly three out of four variables lacked, on average, two responses from an ICTF member. Because we categorized each missing response as a “No”, the relative effect of this decision may have implications for accurately identifying candidate predictors of hospital-induced delirium with any missing responses based on the amount agreement among the ICTF members. While this is an important limitation, it can be corrected by relaxing the criterion for the selection of candidate predictors, for example, using an 78% agreement instead of 100%. We did not do this, however, because we are planning to develop a separate model based on the candidate predictors that reached less than 100% agreement, so we are not discarding any potentially important candidate predictors of hospital-induced delirium. To avoid non-response altogether, a future study may consider designing a survey that would have “forced-entry” answer options, such as is possible in web-based applications for secure data capture.

Second, although we did try to diversify our task force by including members from two different hospitals, one in a rural and another in an urban location, the diversity of our task force may be considered to be limited, because it only included members from the same healthcare system. Clinicians that practice in the same healthcare system may be used to assessing risk of hospital-induced delirium in a different way than clinicians that practice in another healthcare system. A future improvement would be to capture experts’ past work experiences and/or to include experts from different healthcare systems to account for potential differences in the assessment.

Finally, we (temporarily) ignored four variables that were only documented in clinicians’ narrative notes. We also did not consider operationalizing other variables using text data. This is an important limitation, because 32% of clinical data is stored in the form of text, [31] and nurses’ narrative notes are particularly rich in potentially clinically meaningful information about the risk of iatrogenic conditions [32]. To address this limitation, we are conducting a separate study that uses natural language processing methods to identify risk factors of hospital-induced delirium in nurses’ narrative notes (see “Authors’ Information” below the article for more information about the study).

## Conclusions

We described the process of identifying clinical concepts that can be used as candidate predictors to develop a prognostic model of hospital-induced delirium. While we focused on hospital-induced delirium, this process may also be applicable to other iatrogenic conditions. We highlighted the application of a practice-based approach during variable selection by engaging the Iatrogenic Conditions Task Force in the validation of clinical concepts that had been extracted from the literature. The empirical value of this approach needs to be tested in a future study that compares the performance of an expert-driven model against a data-driven model.

### Electronic supplementary material

Below is the link to the electronic supplementary material.

Supplementary Material 1

## Data Availability

All of the data generated or analyzed during this study are included in the published manuscript and its supplementary material.

## Declarations

### Authors’ information (optional)

For more background information about this and the larger UF-ECLIPSE study, go to the National Institutes of Health RePORTER.
