# Supplementary material for: Application of a practice-based approach in variable selection for a prediction model development study of hospital-induced delirium
Source: BMC Med Inform Decis Mak. 2023 Sep 13;23:181. doi: 10.1186/s12911-023-02278-1 (PMC10500854; doi:10.1186/s12911-023-02278-1)
Supplement: Supplementary file 1 — Supplementary Material 1 [file 12911_2023_2278_MOESM1_ESM.pdf]

Supplementary Material

Additional Information about Step 1: Review of Existing Literature

Eligibility Criteria

Original studies were included if they met *each one* of the following inclusion criteria:

1. developed *prognostic* model(s) of hospital-induced delirium for hospitalized adults (a prognostic model is a type of a prediction model that estimates probability that an outcome will occur within a specified time in the future):
    a. primary outcome was the occurrence of hospital-induced delirium (i.e., delirium present: yes or no):
        i. the word "delirium" had to be used for the outcome instead of any similar term, such as "altered mental status", "confusion", or "neurological complication",
        ii. delirium was hospital-induced, i.e., absent on admission;
    b. model(s) was developed using data from the hospitalized adult population:
        i. patients were a minimum of 18 years old,
        ii. patients were hospitalized in inpatient units across the hospital, including medical-surgical units, perioperative units, step-down units, and/or intensive care units;
2. validated their models using any one of the following three ways:
    a. internal validation by:
        i. comparing the Akaike Information Criterion (AIC), Bayesian Information Criterion (BIC), Deviance Information Criterion (DIC), Mallow's Cp, or

adjusted R-squared among various models that were developed using the same set of data (the various models had to be presented in the article or supplementary material),

ii. by bootstrapping or with cross-validation, or by randomly splitting the sample into a training set and test set and developing the model using the training set, and validating the model using the test set;

b. external validation by comparing model performance between an internal dataset (dataset that was used to develop a model) and external dataset (data that was used to validate the model, for example, in a different setting), where both the internal and external datasets came from the same study design.

Studies were excluded if they met *any one* of the following exclusion criteria:

1. did not have delirium as the (primary) outcome:
    a. had delirium as a predictor, for example, in a prognostic model of postoperative complications,
    b. predicted the course of delirium (for example, delirium severity) or outcomes of delirium (for example, post-delirium complications, delirium recovery, delirium survival, etc.), *not* the occurrence of delirium itselt,
2. developed *diagnostic* models of hospital-induced delirium (a diagnostic model is a type of a prediction model that estimates probability that an outcome is present at this time, for example, studies developing or validating delirium assessment tools),
3. failed to validate their prediction models either internally or externally using any one of the three ways that are listed in the inclusion criteria,
4. based in the following settings:

   a. community, including assisted living,
   b. emergency departments/rooms,
   c. gynecologic and/or obstetrical units,
   d. nursing homes/long-term care facilities,
   e. psychiatric hospitals/units
   f. rehabilitation units/outpatient rehabilitation facilities;
5. lacked abstracts for the title and abstract screening or full texts for the full-text screening (including through the interlibrary loan system that is offered by our institution).

Selection Process

The selection process consisted of two parts. The first part involved screening of the records that had been identified in the databases by title and abstract against the eligibility criteria. Five percent of the records were independently screened by two researchers (U.A.S. and T.G.R.M.) and the percentage of agreement was calculated. The researchers then discussed any discrepancies and resolved them via consensus. Any remaining discrepancies were presented to the primary investigator (R.J.L.) for a final resolution. The remaining (unscreened) 95% of the records were halved, and each researcher (U.A.S. and T.G.R.M.) independently reviewed one half. A couple of considerations during this step included: (1) If it was unclear in the abstract whether and/or how the model(s) was validated, the record was tentatively included and assessed for appropriate validation in the full text in part 2 of the selection process, and (2) Literature reviews that seemed relevant were tentatively included and individual records were extracted and screened following step 1 and 2.

The second part of the selection process involved full-text screening of the records that had been included in the title and abstract screening against the eligibility criteria. Both researchers (U.A.S. and T.G.R.M.) independently screened 5% of the full texts. The percentage of agreement was calculated. Any discrepancies were first discussed and then resolved by consensus between the two researchers (U.A.S. and T.G.R.M.). Any unresolved discrepancies were presented to the primary investigator (R.J.L.) for a final resolution. The remainder of the full texts was then halved and each researcher (U.A.S. and T.G.R.M.) independently reviewed one half. The articles that each researcher included were then added and included in the final synthesis.

Figure S1

PRISMA Diagram

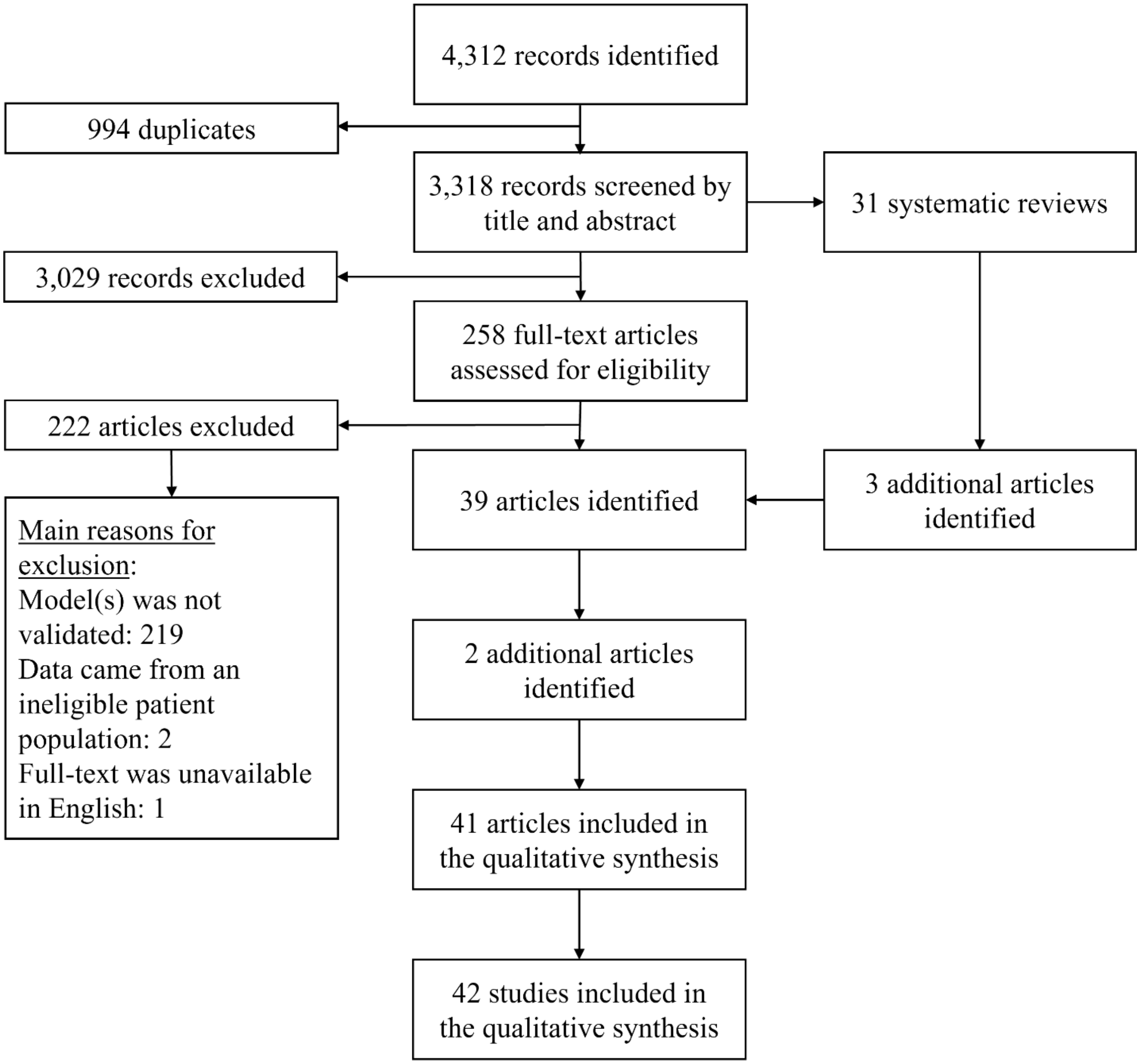

Note. This PRISMA diagram pertains to the systematic review that is described in Step 1. The protocol is available under registration number CRD42020218635 (version 03 December 2020) in the International Prospective Register of Systematic Reviews "PROSPERO". PRISMA = Preferred Reporting Items for Systematic Reviews and Meta-Analyses.

Table S1

Prognostic Model Development-and-Validation Studies of Hospital-induced Delirium (n = 34)

Author Year	Data Source	Study Dates	Sample Size*	Delirium*	No Delirium*	Inclusion Criteria	Statistical Model (Name of the Model, if Applicable)	Sensitivity/ Specificity, Accuracy	AUROC	NPV/PPV	Validation Method
Böhner 2003 [1]	Heinrich Heine University, Germany	09/1997 – 12/1998	153	60	93	Pts undergoing elective arterial surgery w/ anticipated operation time of >90 mins	Logistic regression	BV: 0.810/0.784, 0.795<br>AV: 0.702/0.713, 0.699	-	-	Cross-validation
Carrasco 2014 [2]	University hospital affiliated w/ the Pontifical Catholic University of Chile	-	478	37	441	Pts ≥65 yrs admitted to the general medical ward in the previous 48 hrs	Logistic regression → Linear prediction rule (“delirium predictive risk score”)	0.88/0.74 for the cut-off point of -240	DC: 0.86<br>VC: **0.78**<br>Diff. = -0.08	-	Development and prospective validation cohorts
Chaiwat 2019 [3]	Faculty of Medicine Siriraj Hospital at Mahidol University, Thailand	02/2016 – 02/2017	250	61	189	Pts ≥18 yrs admitted to SICU w/ anticipated stay of >24 hrs within 7 days of surgery	Logistic regression	Multiple reported for different cut-off points	BV: 0.84<br>AV: **0.82**<br>Diff. = -0.02	Multiple reported for different cut-off points	Bootstrapping
Chen 2017 [4]	Lanzhou University Second Hospital, China	05/17/2016 – 09/25/2016	620	160	460	Pts >18 yrs admitted to ICU for ≥24 hrs	Logistic regression	Multiple reported for different risk groups	DS: 0.78<br>VS: **0.78**<br>Diff. = 0.00	Multiple reported for different risk groups	Development and validation sets
Choi 2017 [5]	Samsung Medical Center, South Korea	2003 – 2014	341	89	252	Records of pts who underwent surgical treatment for head and neck cancer w/ free flap reconstruction	Logistic regression → Nomogram	BV: 0.618/0.754<br>AV: 0.449/0.841 for max. Youden’s index	BV: 0.7407<br>AV: **0.6898**<br>Diff. = -0.0509	BV: 0.848/0.470<br>AV: 0.500/0.812 for max. Youden’s index	Cross-validation

Corradi<br>2018 [6]	Hartford Hospital, CT	09/01/2012 –<br>09/30/2015	64,038	3,499	60,539	Records of pts w/ any recorded CAMs and lengths of stay ≥48hrs	Distributed Random Forest	BV:<br>-<br>AV:<br>0.698/0.927	BV:<br>-<br>AV:<br>**0.909**	BV:<br>-<br>AV:<br>-/0.357	Bootstrapping<br><br>Cross-validation<br><br>Training and validation sets
de Wit<br>2016 [7]	Zuyderland Medical Centre, the Netherlands	2008 –<br>2012	1,291	225	1,066	Records of pts ≥60 yrs	Logistic regression (the “medication” model “DEMO”)	BV:<br>0.782/0.637 for the optimal cut-off point of 14.1%<br>AV:<br>-	BV:<br>0.77<br>AV:<br>**0.76**<br>Diff. =<br>-0.01	-	Bootstrapping
Fan<br>2019 [8]	University hospital affiliated with the Capital Medical University, China	01/2009 –<br>01/2010	560	114	446	Pts ≥18 yrs admitted to ICU w/ anticipated stay of ≥24 hrs	Logistic regression → Prediction rule (“DYNAMIC-ICU”)	-	DC BV:<br>0.907<br>DC AV:<br>**0.874**<br>Diff. =<br>-0.033<br>VC:<br>0.900	-	Bootstrapping<br><br>Random split into development and validation cohorts
Hori<br>2014 [9]	Johns Hopkins Hospital, MD	04/2008 –<br>01/2013	491	45	446	Pts undergoing cardiac surgery w/ cardiopulmonary bypass	Logistic regression	-	BV:<br>0.789<br>AV:<br>**0.750**<br>Diff. =<br>-0.039	-	Cross-validation
Inouye<br>1993 [10]	Yale-New Haven Hospital, CT	06/1988 –<br>06/1990	281	56	225	Pts ≥70 yrs admitted to general medicine floor for ≥48 hrs	Proportional hazards model → Risk stratification model	Multiple sensitivities reported for different risk strata	DC:<br>0.74<br>VC:<br>**0.66**<br>Diff. =<br>-0.08	-	Development and prospective validation cohorts
Inouye & Charpentier<br>1996 [11]	Yale-New Haven Hospital, CT	11/06/1989 –<br>07/31/1991	508	82	426	Pts ≥70 yrs admitted to general medicine floor for ≥48 hrs	Binomial regression → Risk stratification model	Multiple sensitivities reported for different risk strata	-	-	Development and prospective validation cohorts
Ji<br>2018 [12]	Hospital in Shanghai, China	03/2016 –<br>04/2017	134	16	118	Pts ≥65 yrs admitted to SICU for ≥24 hrs	Artificial neural network	LS:<br>0.917/0.938<br>TS:<br>0.500/0.784	LS:<br>-<br>TS:<br>**0.893**	LS:<br>0.987/0.688<br>TS:<br>0.936/0.200	Split into learning and testing sets

Jones 2016 [13]	Beth Israel Deaconess Medical Center, MA<br><br>Brigham and Women's Hospital, MA	06/18/2010 – 08/08/2013	566	135	431	Pts ≥70 yrs undergoing major elective non-cardiac surgery w/ anticipated stay of ≥3 days	Logistic regression (the "bivariable model")	-	-	-	Bootstrapping
							Logistic regression (the "multivariable model I")	-	-	-	
							Logistic regression (the "multivariable model II")	-	-	-	
Jung 2018 [14]	Samsung Medical Center, South Korea	04/2010 – 02/2015	980	222	758	Records of pts w/ esophageal cancer who underwent esophagectomy	Logistic regression	-	BV: 0.66<br>AV: **0.63**-0.64<br>Diff. = -0.03	-	Bootstrapping<br><br>Cross-validation
Katznelson 2009 [15]	Toronto General Hospital, Canada	04/2006 – 06/2006	1,059	122	937	Pts undergoing cardiac surgery w/ cardiopulmonary bypass	Logistic regression	-	BV: 0.774<br>AV: **0.746**<br>Diff. = -0.028	-	Bootstrapping
Katznelson 2009 [16]	Toronto General Hospital, Canada	01/2006 – 01/2007	582	128	454	Pts undergoing elective or emergency vascular surgery	Logistic regression	-	BV: 0.746<br>AV: **0.730**<br>Diff. = -0.016	-	Bootstrapping
Kim 2016 [17]	Dongsan Medical Center, South Korea	06/2013 – 10/2014	1,114	211	903	Pts >60 yrs undergoing major surgery w/ anticipated stay of ≥3 days	Logistic regression → Prediction score ("Delphi")	DC:<br>-<br>VC: 0.808/0.925 for the cut-off score of 7	DC: 0.911<br>VC: **0.938**<br>Diff. = +0.027	DC:<br>-<br>VC: 0.957/0.702 for the cut-off score of 7	Development and prospective validation cohorts
Kobayashi 2013 [18]	St. Luke's International Hospital, Japan	04/01/2009 – 03/31/2010	3,570	142	3,428	Records of adult pts admitted to internal medicine unit	Chi-Square Automatic Interaction Detector (CHAID) decision tree	-	DG: 0.82<br>VG: **0.82**<br>Diff. = 0.00	-	Random split into development and validation groups

							Logistic regression	-	DG: 0.78 VG: **0.79** Diff. = +0.01	-	
Kostalova 2012 [19]	Hospital of the Masaryk University, Czech Republic	01/2009 – 03/2010	100	43	57	Pts admitted to stroke unit	Logistic regression	BV: - AV: 0.690/0.842	-	-	Cross-validation
							Logistic regression (the “alternative” model)	BV: - AV: 0.651/0.807	-	-	
Kumar 2017 [20]	Postgraduate Institute of Medical Education and Research, India	-	120	21	99	Adults 18-80 yrs undergoing elective or emergency cardiac surgery	Logistic regression	-	-	-	Bootstrapping
Leung 2007 [21]	University of California San Francisco Medical Center, CA	2001 – 2006	190	29	161	Pts ≥65 yrs undergoing major elective non-cardiac surgery w/ anticipated stay of >48 hrs	Logistic regression	-	-	-	Bootstrapping
Levkoff 1988 [22]	Beth Israel Hospital, MA	1984 – 1986	1,756	160	1,596	Records of pts ≥60 yrs w/ stay >2 days	Recursive partitioning	-	-	-	Development and prospective validation series
Neefjes 2017 [23]	VUmc Cancer Center Amsterdam, the Netherlands	01/01/2011 – 09/2013	620	98	522	Records of pts w/ solid malignancies admitted to medical oncology ward	Tree analysis	BV: - AV: 0.4/0.85	BV: 0.81 AV: **0.65** Diff. = -0.16	-	Cross-validation
O’Keeffe & Lavan 1996 [24]	Royal Liverpool University Hospital, the UK	-	184	53	131	Pts admitted to acute-care geriatric unit w/ anticipated stay of ≥48 hrs	Logistic regression → Risk stratification model	Multiple sensitivities reported for different risk strata	DG: 0.79 VG: **0.75** Diff. = -0.04	-	Derivation and prospective validation groups

Pisani 2007 [25]	Yale-New Haven Hospital, CT	09/05/2002 – 09/30/2004	304	214	90	Pts ≥60 yrs admitted to medical ICU	Logistic regression	-	Mean **0.78**	-	Bootstrapping
Pompei 1994 [26]	University of Chicago Hospitals, IL<br><br>Yale-New Haven Hospital, CT	11/1989 – 06/1991	755	150	605	Pts ≥65 yrs (derivation set) or ≥70 yrs (test set) admitted to medical/surgical ward for ≥48 hrs	Logistic regression → Risk stratification model	Multiple sensitivities reported for different risk strata	DS: 0.74 TS: **0.64** Diff. = -0.10	-	Derivation and prospective test sets
Roijers 2020 [27]	Amphia Hospital, the Netherlands	01/2013 – 06/2018	392	70	322	Records of pts ≥65 yrs undergoing surgical or endovascular treatment for critical limb ischemia w/ stay of ≥2 days	Logistic regression	-	BV: 0.82 AV: **0.82** Diff. = 0.00	-	Cross-validation
Rudolph 2009 [28]	Two academic medical centers, U.S.<br><br>One Veterans Affairs hospital, U.S.	09/01/2002 – 06/30/2006	231	111	120	Pts >60 yrs undergoing cardiac surgery	Logistic regression → prediction rule	Multiple sensitivities reported for different rule points	DS: 0.74 VS: **0.75** Diff. = +0.01	-	Bootstrapping<br><br>Derivation and prospective validation sets
Tse 2015 [29]	St. Paul's Hospital, Canada	01/01/2008 – 12/31/2008	679	190	489	Patients undergoing cardiac surgery	Logistic regression	-	**0.732** (no before/ after)	-	Bootstrapping
van den Boogaard 2012 [30]	Radboud University Nijmegen Medical Center, the Netherlands	02/01/2008 – 09/2009	2,162	582	1,580	Adult pts w/ complete CAM-ICU screenings admitted to ICU for ≥1 day	Logistic regression ("PRE-DELIRIC")	Multiple reported	DC BV: 0.87 DC AV: **0.86** Diff. = -0.01 VC: 0.89	-	Bootstrapping<br><br>Development and prospective validation cohorts
Wassenaar 2015 [31]	University/ university affiliated	10/2011 – 06/2012	2,914	689	2,225	Pts ≥18 yrs w/ complete CAM-ICU screenings	Logistic regression ("E-	Multiple reported	DC: 0.76 VC:	-	Development and prospective

	hospitals in Australia, Belgium, England, Germany, Spain, Sweden, and the Netherlands					admitted to ICU for ≥1 day	PRE-DELIRIC")		**0.75** Diff. = -0.01		validation cohorts
Wong 2018 [32]	University of California San Francisco Health, CA	01/01/2016 – 11/30/2017	18,223	878	17,345	Records of adult patients w/ ≥1 CAM-ICU or Nu-DESC score	Penalized logistic regression	Multiple reported	**0.854**	Multiple reported	Cross-validation
							Gradient boosting machine	Multiple reported	**0.855**	Multiple reported	Training and test sets
							Artificial neural network	-	-	-	
							Linear support vector machine	-	-	-	
							Random forest	Multiple reported	**0.848**	Multiple reported	
Xing 2019 [33]	University hospital in Heilongjiang Province, China	04/2017 – 01/2018	400	112	288	Patients ≥18 yrs admitted to ICU after surgery for at ≥24 hrs	Logistic regression	Multiple reported for different cut-off points	**0.852** (no before/ after)	-	Development and validation sets
Zhan 2020 [34]	The First Affiliated Hospital of the University of Science and Technology of China, China	01/2015 – 08/2019	229	47	182	Pts ≥50 yrs undergoing deep brain stimulation for primary Parkinson's disease	Logistic regression → Nomogram	BV: 0.745/0.731 AV: 0.745/0.731	BV: 0.769 AV: **0.755** Diff. = -0.014	BV: 0.917/0.417 AV: 0.917/0.417	Bootstrapping

Note. The studies are ordered alphabetically by the first author's last name. Post-validation values of AUROC in model development and validation studies are bolded. AUROC = area under the receiver operating curve; AV = after validation; BV = before validation; CAM = Confusion Assessment Method; CAM-ICU = Confusion Assessment Method Intensive Care Unit; DC = development cohort; DG = development group; Diff. = difference; DS = development/derivation set; ICDSC = Intensive Care Delirium Screening Checklist; ICU = intensive care unit; NPV = negative predictive value; NuDESC = Nursing Delirium Screening Scale; PPV = positive predictive value; pts = patients; SICU = surgical intensive care unit; TS = test set; VC = validation cohort; VG = validation group; VS = validation set; yrs = years.

* Total number from both cohorts for studies with the development and prospective validation cohorts.

References:

1. Böhner H, Hummel TC, Habel U, et al. Predicting delirium after vascular surgery: A model based on pre- and intraoperative data. Ann Surg. 2003;238(1):149-156. doi:10.1097/01.sla.0000077920.38307.5f
2. Carrasco MP, Villarroel L, Andrade M, Calderón J, González M. Development and validation of a delirium predictive score in older people. Age Ageing. 2014;43(3):346-351. doi:10.1093/ageing/aft141
3. Chaiwat O, Chanidnuan M, Pancharoen W, et al. Postoperative delirium in critically ill surgical patients: Incidence, risk factors, and predictive scores. BMC Anesthesiol. 2019;19(1):39. doi:10.1186/s12871-019-0694-x
4. Chen Y, Du H, Wei BH, Chang XN, Dong CM. Development and validation of risk-stratification delirium prediction model for critically ill patients: A prospective, observational, single-center study. Medicine (Baltimore). 2017;96(29):e7543. doi:10.1097/MD.0000000000007543
5. Choi NY, Kim EH, Baek CH, Sohn I, Yeon S, Chung MK. Development of a nomogram for predicting the probability of postoperative delirium in patients undergoing free flap reconstruction for head and neck cancer. Eur J Surg Oncol. 2017;43(4):683-688. doi:10.1016/j.ejso.2016.09.018
6. Corradi JP, Thompson S, Mather JF, Waszynski CM, Dicks RS. Prediction of incident delirium using a random forest classifier. J Med Syst. 2018;42(12):261. doi:10.1007/s10916-018-1109-0
7. de Wit HA, Winkens B, Mestres Gonzalvo C, et al. The development of an automated ward independent delirium risk prediction model. Int J Clin Pharm. 2016;38(4):915-923. doi:10.1007/s11096-016-0312-7
8. Fan H, Ji M, Huang J, et al. Development and validation of a dynamic delirium prediction rule in patients admitted to the intensive care units (DYNAMIC-ICU): A prospective cohort study. Int J Nurs Stud. 2019;93:64-73. doi:10.1016/j.ijnurstu.2018.10.008
9. Hori D, Brown C, Ono M, et al. Arterial pressure above the upper cerebral autoregulation limit during cardiopulmonary bypass is associated with postoperative delirium. Br J Anaesth. 2014;113(6):1009-1017. doi:10.1093/bja/aeu319
10. Inouye SK, Viscoli CM, Horwitz RI, Hurst LD, Tinetti ME. A predictive model for delirium in hospitalized elderly medical patients based on admission characteristics. Ann Intern Med. 1993;119(6):474-481. doi:10.7326/0003-4819-119-6-199309150-00005
11. Inouye SK, Charpentier PA. Precipitating factors for delirium in hospitalized elderly persons. Predictive model and interrelationship with baseline vulnerability. JAMA. 1996;275(11):852-857.
12. Ji MT, Xing SP, Yang Y. Pathophysiological factors of delirium among critically ill elders after non-cardiac surgery based on artificial neural networks: A pilot study. Anaesth, Pain & Intensive care. 2018;22(4):424-430.
13. Jones RN, Marcantonio ER, Saczynski JS, et al. Preoperative cognitive performance dominates risk for delirium among older adults. J Geriatr Psychiatry Neurol. 2016;29(6):320-327. doi:10.1177/0891988716666380
14. Jung DM, Ahn HJ, Yang M, et al. Hydroxyethyl starch is associated with early postoperative delirium in patients undergoing esophagectomy. J Thorac Cardiovasc Surg. 2018;155(3):1333-1343. doi:10.1016/j.jtcvs.2017.10.077
15. Katznelson R, Djaiani GN, Borger MA, et al. Preoperative use of statins is associated with reduced early delirium rates after cardiac surgery. Anesthesiology. 2009;110(1):67-73. doi:10.1097/ALN.0b013e318190b4d9
16. Katznelson R, Djaiani G, Mitsakakis N, et al. Delirium following vascular surgery: Increased incidence with preoperative beta-blocker administration. Can J Anaesth. 2009;56(11):793-801. doi:10.1007/s12630-009-9148-0
17. Kim MY, Park UJ, Kim HT, Cho WH. Delirium prediction based on hospital information (Delphi) in general surgery patients. Medicine (Baltimore). 2016;95(12):e3072. doi:10.1097/MD.0000000000003072
18. Kobayashi D, Takahashi O, Arioka H, Koga S, Fukui T. A prediction rule for the development of delirium among patients in medical wards: Chi-Square Automatic Interaction Detector (CHAID) decision tree analysis model. Am J Geriatr Psychiatry. 2013;21(10):957-962. doi:10.1016/j.jagp.2012.08.009
19. Kostalova M, Bednarik J, Mitasova A, et al. Towards a predictive model for post-stroke delirium. Brain Inj. 2012;26(7-8):962-971. doi:10.3109/02699052.2012.660510
20. Kumar AK, Jayant A, Arya VK, Magoon R, Sharma R. Delirium after cardiac surgery: A pilot study from a single tertiary referral center. Ann Card Anaesth. 2017;20(1):76-82. doi:10.4103/0971-9784.197841
21. Leung JM, Sands LP, Wang Y, et al. Apolipoprotein E e4 allele increases the risk of early postoperative delirium in older patients undergoing noncardiac surgery. Anesthesiology. 2007;107(3):406-411. doi:10.1097/01.anes.0000278905.07899.df
22. Levkoff SE, Safran C, Cleary PD, Gallop J, Phillips RS. Identification of factors associated with the diagnosis of delirium in elderly hospitalized patients. J Am Geriatr Soc. 1988;36(12):1099-1104. doi:10.1111/j.1532-5415.1988.tb04396.x

23. Neefjes ECW, van der Vorst MJDL, Verdegaal BATT, Beekman ATF, Berkhof J, Verheul HMW. Identification of patients with cancer with a high risk to develop delirium. Cancer Med. 2017;6(8):1861-1870. doi:10.1002/cam4.1106
24. O'Keeffe ST, Lavan JN. Predicting delirium in elderly patients: Development and validation of a risk-stratification model. Age Ageing. 1996;25(4):317-321. doi:10.1093/ageing/25.4.317
25. Pisani MA, Murphy TE, Van Ness PH, Araujo KL, Inouye SK. Characteristics associated with delirium in older patients in a medical intensive care unit. Arch Intern Med. 2007;167(15):1629-1634. doi:10.1001/archinte.167.15.1629
26. Pompei P, Foreman M, Rudberg MA, Inouye SK, Braund V, Cassel CK. Delirium in hospitalized older persons: Outcomes and predictors. J Am Geriatr Soc. 1994;42(8):809-815. doi:10.1111/j.1532-5415.1994.tb06551.x
27. Roijers JP, Rakké YS, Hopmans CJ, et al. Incidence and risk factors for delirium in elderly patients with critical limb ischaemia. Eur J Vasc Endovasc Surg. 2020;59(4):598-605. doi:10.1016/j.ejvs.2019.11.029
28. Rudolph JL, Jones RN, Levkoff SE, et al. Derivation and validation of a preoperative prediction rule for delirium after cardiac surgery. Circulation. 2009;119(2):229-236. doi:10.1161/CIRCULATIONAHA.108.795260
29. Tse L, Schwarz SK, Bowering JB, Moore RL, Barr AM. Incidence of and risk factors for delirium after cardiac surgery at a quaternary care center: A retrospective cohort study. J Cardiothorac Vasc Anesth. 2015;29(6):1472-1479. doi:10.1053/j.jvca.2015.06.018
30. van den Boogaard M, Pickkers P, Slooter AJ, et al. Development and validation of PRE-DELIRIC (prediction of delirium in ICU patients) delirium prediction model for intensive care patients: Observational multicentre study. BMJ. 2012;344:e420. doi:10.1136/bmj.e420
31. Wassenaar A, van den Boogaard M, van Achterberg T, et al. Multinational development and validation of an early prediction model for delirium in ICU patients. Intensive Care Med. 2015;41(6):1048-1056. doi:10.1007/s00134-015-3777-2
32. Wong A, Young AT, Liang AS, Gonzales R, Douglas VC, Hadley D. Development and validation of an electronic health record-based machine learning model to estimate delirium risk in newly hospitalized patients without known cognitive impairment. JAMA Netw Open. 2018;1(4):e181018. doi:10.1001/jamanetworkopen.2018.1018
33. Xing H, Zhou W, Fan Y, Wen T, Wang X, Chang G. Development and validation of a postoperative delirium prediction model for patients admitted to an intensive care unit in China: A prospective study. BMJ Open. 2019;9(11):e030733. doi:10.1136/bmjopen-2019-030733
34. Zhan L, Wang XQ, Zhang LX. Nomogram model for predicting risk of postoperative delirium after deep brain stimulation surgery in patients older than 50 years with Parkinson disease. World Neurosurg. 2020;139:e127-e135. doi:10.1016/j.wneu.2020.03.160

Table S2

CHARMS Checklist: Candidate Predictors (Or Index Tests)

Author<br>Year	Number and type of predictors (e.g., demographics, patient history, physical examination, additional testing, disease characteristics)	Definition and method for measurement of candidate predictors	Timing of predictor measurement (e.g., at patient presentation, at diagnosis, at treatment initiation)	Were predictors assessed blinded for outcome, and for each other (if relevant)?	Handling of predictors in the modelling (e.g., continuous, linear, non-linear transformations or categorized)
Böhner<br>2003 [1]	✓	✓	✓		✓
Carrasco<br>2014 [2]	✓	✓	✓		✓
Chaiwat<br>2019 [3]	✓	✓	✓		✓
Chen<br>2017 [4]	✓		✓		✓
Choi<br>2017 [5]	✓	✓	✓		✓
Corradi<br>2018 [6]	✓	✓	✓		✓
de Wit<br>2016 [7]	✓	✓	✓		✓
Fan<br>2019 [8]	✓	✓	✓		✓
Hori<br>2014 [9]	✓	✓	✓		✓
Inouye<br>1993 [10]	✓	✓	✓		✓
Inouye & Charpentier<br>1996 [11]	✓	✓	✓	✓	✓
Ji<br>2018 [12]	✓		✓		✓
Jones<br>2016 [13]	✓	✓	✓	✓	✓
Jung<br>2018 [14]	✓	✓	✓		✓
Katznelson<br>2009 [15]	✓	✓	✓		✓
Katznelson<br>2009 [16]	✓	✓	✓		✓

Kim<br>2016 [17]	✓		✓		✓
Kobayashi<br>2013 [18]	✓	✓	✓		✓
Kostalova<br>2012 [19]	✓	✓	✓		✓
Kumar<br>2017 [20]	✓	✓	✓		✓
Leung<br>2007 [21]	✓	✓	✓		✓
Levkoff<br>1988 [22]	✓	✓	✓		✓
Neefjes<br>2017 [23]	✓	✓			✓
O'Keeffe & Lavan<br>1996 [24]	✓	✓	✓		✓
Pisani<br>2007 [25]	✓	✓	✓	✓	✓
Pompei<br>1994 [26]	✓	✓	✓		✓
Roijers<br>2020 [27]	✓	✓			✓
Rudolph<br>2009 [28]	✓	✓	✓		✓
Tse<br>2015 [29]	✓				✓
van den Boogaard<br>2012 [30]	✓	✓	✓		✓
Wassenaar<br>2015 [31]	✓	✓	✓		✓
Wong<br>2018 [32]	✓	✓	✓		✓
Xing<br>2019 [33]	✓	✓	✓		✓
Zhan<br>2020 [34]	✓	✓	✓		✓

Note. This table shows the "Candidate Predictors (Or Index Tests)" domain only. CHARMS = Checklist for Critical Appraisal and Data Extraction for Systematic Reviews of Prediction Modelling Studies. See Table S1 for the references.

Box S1

Instructions to the ICTF Members*

"We would like your help to identify variables that are clinically relevant to hospital-induced delirium. Please review the attached Excel spreadsheet and consider each term based on your expert knowledge and clinical judgement. Select YES if you think the term is associated with hospital-induced delirium or NO if you do not think the term is associated with hospital-induced delirium. If you think of any other clinical terms that are associated with hospital-induced delirium, please include them at the bottom of the spreadsheet in the designated cell."

Note. ICTF = Iatrogenic Conditions Task Force.
* These instructions were sent to the ICTF members in an e-mail with the attached Excel spreadsheet.

Table S3

Variables Extracted from the Literature Review (n = 504)*

Variable	Number of ICTF Members who Judged the Variable to Be Associated with the Development of Hospital-Induced Delirium (out of 9)
Age	9
Agitation/Sedation	9
Alcohol use	9
Alzheimer's disease	9
Anxiety	9
Cerebral edema	9
Cognitive status	9
Dementia	9
Depression	9
Duration of anesthesia	9
Fentanyl	9
General anesthesia	9
History of delirium	9
Length of hospital stay	9
Length of intensive care unit stay	9
Mental disorder	9
Minerals and electrolytes	9
Neurologic disease	9
Neurosurgery	9
NPO state	9
Opioids	9
Pain	9
Physical restraints	9
Physical status	9
Post-surgical complications	9
Prolonged bleeding (due to procedure and/or overanticoagulation)	9
Propofol	9

Psychological status	9
Psychosis	9
Psychotherapeutic agents	9
Respiratory disease	9
Respiratory failure	9
Respiratory infection	9
Risk of falls	9
Sedatives	9
Sepsis	9
Severity of acute illness	9
Severity of stroke	9
Shock	9
Sleep deprivation	9
Sleep disorder	9
Substance use	9
Surgery	9
Trauma	9
Trauma surgery	9
Urinary tract infection	9
Withdrawal	9
Ammonia	8
Antidepressants	8
Artificial ventilation	8
Blood loss	8
Burden of comorbidity	8
Cardiogenic shock	8
Cerebral atrophy	8
Cerebral infarction	8
Cerebral ischemia	8
Coma	8
Dehydration	8
Delirium risk	8
Duration of surgery	8
General anesthetics	8
General surgery	8
History of cerebrovascular accident/transient ischemic attack	8
Hydromorphone	8
Hypoxemia	8
Infection	8
Liver disease	8
Liver failure	8
Living in nursing home	8
Mechanical ventilation	8
Mobility	8
Mood disorder	8
Morphine	8
Nutritional status	8

Open surgery	8
Patient-controlled analgesia	8
pH	8
Polypharmacy	8
Pressure injury	8
Pulmonary disease	8
Respiratory acidosis	8
Respiratory alkalosis	8
Respiratory insufficiency	8
Severity of cirrhosis	8
Stroke	8
Trauma admission	8
Urinary retention	8
Acute kidney disease	7
Admission to post-anesthesia care unit	7
Admission to the intensive care unit	7
Any iatrogenic event	7
Aphasia	7
Aspiration	7
Benzodiazepines	7
Blood urea nitrogen to creatinine ratio	7
Cancer stage	7
Cardiac surgery	7
Cerebral oxygen saturation	7
Cirrhosis	7
Creatinine	7
Days since onset of pain	7
Electrolytic, caloric, and water balance	7
End-stage renal disease	7
Foley catheter	7
Hearing impairment	7
Hepatitis	7
History of coma	7
History of falls	7
Insomnia	7
Intracranial tumor	7
Ketamine	7
Location of stroke	7
Metabolic acidosis	7
Metabolic alkalosis	7
Metastasis	7
Mobility	7
Neurological/neurosurgical admission	7
Opiate antagonists	7
Oxygen device_BiPAP	7
Oxygen device_CPAP	7
Oxygen device_high flow nasal cannula	7

Oxygen device_non-rebreather mask	7
Oxygen device_trach mask	7
Pancreatic and biliary surgery	7
Parkinson's disease	7
Pneumonia	7
Respiratory rate	7
Severity of Parkinson's disease	7
Sodium	7
Systemic inflammatory response syndrome	7
Temperature	7
Thoracic surgery	7
Tracheostomy	7
Tricyclic/tetracyclic antidepressants	7
Tube feeding	7
Type of intensive care unit	7
Type of stroke (infarction or hemorrhage)	7
Type of surgery (elective or emergency)	7
Visual impairment	7
Amount of blood transfused	6
Analgesics	6
Anorexigenic agents and respiratory and cerebral stimulants	6
Anticholinergic agents	6
Anti-dementia medications	6
Antimanic agents	6
Antiparkinson medications	6
Antipsychotics	6
Arterial partial pressure of oxygen to fraction of inspired oxygen ratio	6
Blood pressure	6
Blood urea nitrogen	6
Bowel or bladder habits	6
Combined general/epidural anesthesia	6
Combined general/regional anesthesia	6
Diabetes mellitus	6
Direct bilirubin	6
Diuretics	6
Duration of mechanical ventilation	6
Fluid intake	6
Fracture	6
Functional status	6
Glucose	6
Head or neck surgery	6
Hematocrit	6
Hemineglect	6
Hemiparesis	6
Hemoglobin	6
Hepatic surgery	6

Hip or knee replacement surgery	6
History of central nervous system disorders	6
History of major amputation	6
Hypnotics	6
Hypotension	6
Hypotensive agents	6
Intraaortic balloon pump use	6
Lactate	6
Location of tumor	6
Lorazepam	6
Malignancy	6
Nasogastric tube	6
Nonbenzodiazepine hypnotics	6
Nonhealing wound	6
Number of intravenous catheters	6
Oxygen device_face tent	6
Oxygen device_simple mask	6
Paralysis	6
Partial pressure of carbon dioxide, arterial	6
Partial pressure of oxygen, arterial	6
Peripheral oxygen saturation (SpO2)	6
Phenylephrine	6
Pneumothorax	6
Sedimentation rate	6
Serum bicarbonate level	6
Skeletal muscle relaxants	6
Social support	6
Spine surgery	6
Surgical admission	6
Surgical risk	6
Thoracic trauma	6
Toileting	6
Total bilirubin	6
Total parenteral nutrition	6
Type of admission (elective vs. emergency)	6
Urea	6
Vasopressors	6
White blood cell count	6
Albumin	5
Alkaline phosphatase	5
Anion gap	5
Anticonvulsants	5
Antihistamines	5
Antiinfective agents	5
Anxiolytics	5
Aortic valve replacement/mitral valve replacement	5
Autonomic drugs, miscellaneous	5

Base excess	5
Blood (red blood cells) transfusion	5
Blood formation, coagulation, and thrombosis	5
Carcinomatous meningitis	5
Central venous pressure	5
Chest tube	5
Chronic kidney disease	5
Code status	5
Coronary artery bypass graft	5
Coronary artery bypass graft + aortic valve replacement/mitral valve replacement	5
Corticosteroids	5
Diastolic blood pressure	5
Difficulty chewing	5
Dopamine	5
Dysuria	5
Elimination	5
Esophagocolonogastrostomy	5
Etiology of stroke	5
Feeding	5
Gastric surgery	5
Gastrointestinal hemorrhage	5
Glomerular filtration rate	5
Heart failure	5
Hemothorax	5
History of femoral neck fracture	5
Human immunodeficiency virus	5
Hypertension	5
Hypothyroidism	5
Immobilizing device	5
In/Out of bed	5
Interstitial lung disease	5
Intravenous cathether complications	5
Living alone	5
Mean arterial pressure	5
Midazolam	5
Neuroleptics	5
Oxygen device_aerosol mask	5
Oxygen device_T-piece	5
Oxytocics	5
Pharmaceutical aids	5
Pneumonitis	5
Potassium	5
Prealbumin	5
Prolonged emergency department stay	5
Readmission	5
Red blood cell count	5
Reoperation	5

Respiratory tract agents	5
Revascularization of lower extremities	5
Selective serotonin reuptake inhibitors	5
Serotonin antagonist and reuptake inhibitors	5
Serotonin-norepinephrine reuptake inhibitors	5
Smooth muscle relaxants	5
Sympathomimetic (adrenergic) agents	5
Thyroid disease	5
Total protein	5
Transfusion reaction	5
Tumor	5
Urine protein	5
Urine specific gravity	5
Urine white blood cell count	5
Urologic surgery	5
Vascular surgery	5
Volume of stroke	5
Alanine transaminase	4
Aminoketones	4
Amputation	4
Amylase	4
Antidiabetics	4
Antiemetics	4
Antimigraine agents	4
Antineoplastic agents	4
Aortic cross clamp	4
Aortic reconstructive surgery	4
Aortic root	4
Aortoiliacal occlusive disease	4
Blood derivatives	4
Braces/devices/sensory aids_external fixator	4
Braces/devices/sensory aids_halo	4
Bronchodilators	4
Burden of tumor	4
Cardiac drugs	4
Carotid artery disease	4
Coagulopathy	4
Colorectal surgery	4
Congenital cyanotic heart disease	4
Cor pulmonale	4
Debridement	4
Digoxin (level)	4
Duration of aortic cross clamp	4
Duration of cardiopulmonary bypass	4
Ear, nose, and throat surgery	4
Embolectomy	4
Endovascular aortic repair	4

Endovascular revascularization	4
Enzymes	4
Etomidate	4
Exploratory laparotomy	4
Fibromyalgia agents	4
First generation antihistamines	4
Fresh frozen plasma transfusion	4
Grooming	4
Gynecologic surgery	4
Histamine-2 blockers	4
History of myocardial infarction	4
History of vascular disease	4
History of vascular surgery	4
Infrarenal abdominal aortic aneurysm	4
Ischemic cardiac disease	4
Laxatives	4
Left ventricular assisting device placement	4
Left ventricular ejection fraction	4
Level of education	4
Literacy	4
Local anesthesia with conscious sedation	4
Lymphoma	4
Neoadjuvant therapy	4
Noninvasive ventilation	4
Ondansetron	4
Ophthalmics	4
Oxygen device_blow-by	4
Partial thromboplastin time	4
Patient-controlled epidural analgesia	4
Peripheral arterial occlusive disease	4
Peripheral vascular disease	4
Platelet count	4
Pulmonary hypertension	4
Regional anesthesia	4
Relieving factors_medication	4
Rocuronium	4
Second generation antihistamines	4
Sevoflurane	4
Smoking status	4
Sufentanil	4
Surgical revascularization	4
Surgical site	4
Sympatholytic (adrenergic blocking) agents	4
Systolic blood pressure	4
Thiopentone/sodium thiopental	4
Thoracoabdominal aortic aneurysm	4
Thrombectomy	4

Total gastrectomy	4
Total iron binding capacity	4
Transhiatal surgery	4
Unintentional injury	4
Vancomycin	4
Volume overload	4
Weakness of arms/hands	4
Weakness of legs	4
3-Field surgery	3
Abdominal surgery	3
Admission category	3
Albumin (fluid)	3
Alpha blockers	3
Antiasthmatics	3
Antidotes	3
Antipyretics	3
Arrhythmia	3
Aspartate transaminase	3
Asthma	3
Atrial fibrillation	3
Atropine	3
Braces/devices/sensory aids_helmet	3
Braces/devices/sensory aids_immobilizer	3
Braces/devices/sensory aids_sensory aids	3
Braces/devices/sensory aids_splint	3
Bronchiectasis	3
Calcium	3
Cardiac disease	3
Chloride	3
Chronic obstructive pulmonary disease	3
Congenital acyanotic heart disease	3
Constipation	3
Desflurane	3
Devices	3
Dietitian consult needed	3
Digoxin	3
Epidural	3
Extent of peripheral vascular disease	3
Heart rate	3
History of cancer treatment	3
History of carotid endarterectomy	3
History of coronary artery bypass graft	3
History of femoral endarterectomy	3
History of minor amputation	3
History of percutaneous coronary intervention	3
Home nurse visits	3
Inotropes	3

Ivor–Lewis surgery	3
Marital status	3
Mean corpuscular volume of red blood cells	3
Medical admission	3
Minimally invasive surgery	3
Misc. antiinfectives	3
Miscellaneous therapeutic agents	3
Off-pump surgery	3
Oxygen device_room air	3
Phosphate	3
Plastic surgery	3
Platelet transfusion	3
Protamine	3
Relieving factors_cold	3
Relieving factors_distractions	3
Remifentanil	3
Renin-angiotensin-aldosterone system inhibitors	3
Rheumatic disease	3
Serums, toxoids, vaccines	3
Sex	3
Sleep habit details_lights off	3
Sleep habit details_sleep medicine	3
Sleep habit details_television	3
Supraaortic occlusive disease	3
Tube	3
Valvular heart disease	3
Vasodilating agents	3
3-Hole surgery	2
Admission department (unit)	2
Admission service	2
Admission source	2
Angina	2
Bathing	2
Beta blockers	2
Braces/devices/sensory aids_abdominal binder	2
Braces/devices/sensory aids_abductor pillow	2
Braces/devices/sensory aids_brace	2
Braces/devices/sensory aids_chest binder	2
Braces/devices/sensory aids_collar	2
Braces/devices/sensory aids_orthotics	2
Braces/devices/sensory aids_prosthesis	2
Braces/devices/sensory aids_sling	2
Calcium channel blocking agents	2
Cefazolin	2
Cephalosporins	2
Chest drain volume	2
Colloid	2

Coronary artery disease	2
C-reactive protein	2
Crystalloid	2
Dexmedetomidine	2
Diagnostic agents	2
Forced expiratory volume in 1 second (FEV1)	2
Gamma-glutamyl transferase	2
Gastrointestinal drugs	2
Heparin	2
History of cardiac surgery	2
Hormones and synthetic substitutes	2
Intravenous catheter	2
Local anesthetics	2
Medicaid status	2
Nasal agents, systemic and topical	2
Nonsteroidal antiinflammatory drugs	2
Percentage carbohydrate-deficient transferrin (%CDT)	2
Prothrombin time	2
Relieving factors_heat	2
Relieving factors_massage	2
Relieving factors_none	2
Relieving factors_other	2
Relieving factors_relaxation	2
Relieving factors_reposition	2
Relieving factors_spiritual care	2
Skin and mucous membrane agents	2
Sleep habit details_ear plugs	2
Sleep habit details_eye mask	2
Sleep habit details_fan	2
Sleep habit details_music	2
Statins	2
Tranexamic acid	2
Ulcer medications	2
Vitamins	2
Weight loss	2
Angiotensin-converting enzyme inhibitors	1
Ankle-brachial index	1
Antilipemic agents	1
Apolipoprotein 4	1
Body mass index	1
Body weight	1
Braces/devices/sensory aids_Pavlik harness	1
Dressing	1
Eye, ear, nose, and throat preparations	1
Hydroxyethyl starch	1
Hypercholesterinemia	1
Hyperlipidemia	1

Occupation	1
Payor	1
Peptic ulcer disease	1
Race	1
Religion	1
Antihyperlipidemics	0
Body length	0
Ethnicity	0

[*] The literature review included 34 prognostic model development-and-validation studies of hospital-induced delirium (see Table S1).

Note. ICTF = Iatrogenic Conditions Task Force.
